# Trends of survival in patients with multiple myeloma in Japan: a multicenter retrospective collaborative study of the Japanese Society of Myeloma

**DOI:** 10.1038/bcj.2015.79

**Published:** 2015-09-18

**Authors:** S Ozaki, H Handa, T Saitoh, H Murakami, M Itagaki, H Asaoku, K Suzuki, A Isoda, M Matsumoto, M Sawamura, J Konishi, K Sunami, N Takezako, S Hagiwara, Y Kuroda, T Chou, E Nagura, K Shimizu

**Affiliations:** 1 Department of Hematology, Tokushima Prefectural Central Hospital, Tokushima, Japan; 2 Department of Hematology, Gunma University, Maebashi, Japan; 3 Department of Laboratory Sciences, Gunma University Graduate School of Health Sciences, Maebashi, Japan; 4 Department of Hematology, Hiroshima Red Cross Hospital, Hiroshima, Japan; 5 Department of Hematology, Japanese Red Cross Medical Center, Tokyo, Japan; 6 Department of Hematology, National Hospital Organization Nishigunma National Hospital, Shibukawa, Japan; 7 Department of Hematology, National Hospital Organization Okayama Medical Center, Okayama, Japan; 8 Division of Hematology, National Hospital Organization Disaster Medical Center, Tokyo, Japan; 9 Department of Hematology, National Center for Global Health and Medicine, Tokyo, Japan; 10 Department of Hematology, Hiroshima University Hospital, Hiroshima, Japan; 11 Department of Internal Medicine, Niigata Cancer Center Hospital, Niigata, Japan; 12 Department of Hematology, Chutoen General Medical Center, Kakegawa, Japan; 13 Department of Hematology, Tokai Central Hospital, Kakamigahara, Japan

Multiple myeloma (MM) is a heterogeneous disease in the context of treatment outcomes depending on the presence or absence of the disease-related risk factors including stage and chromosomal abnormality, patient-related factors including age and comorbidity and treatment-related factors including the use of novel agents and/or autologous stem cell transplantation (ASCT).^1^ The prevalence of MM varies according to ethnicity and geographical backgrounds, and the incidence of MM in Asia has been reported to be lower than that in Western countries. However, recent reports have shown an increase in the morbidity in Asia.^2^ In Japan, during 1975–2010 the age-adjusted incidence of MM increased from 0.92 to 5.2 and from 0.81 to 4.8 per 100 000 men and women, respectively.^3^

Clinical trials of novel agents comprising thalidomide, lenalidomide and bortezomib have shown a significant improvement in progression-free survival and overall survival (OS) in both transplant-eligible and ineligible patients.^4, 5^ Moreover, supportive treatment with bisphosphonates has shown a positive effect on OS.^6^ Several population-based studies and surveillances have attributed such a recent progress to the introduction of novel agents as well as ASCT.^7, 8, 9, 10^ However, the clinical benefit of such emerged treatment remains unveiled in routine practice in Japan.

Previously, the Japanese Society of Myeloma surveyed the clinical features of 1383 patients diagnosed and treated between January 1990 and December 2000.^11^ At that time, neither proteasome inhibitors nor immunomodulatory drugs were available for the routine practice in Japan, and most patients were treated with conventional chemotherapy alone and the remaining patients were treated with ASCT after induction with conventional chemotherapeutic agents. In Japan, bortezomib was the first novel agent approved for relapsed and/or refractory MM in 2006, and thalidomide and lenalidomide followed in 2008 and 2010, respectively. We hereby conducted a multicenter retrospective study to evaluate the change in survival according to treatment modalities in routine practice in Japan.

Clinical data of 2234 patients newly diagnosed between January 2001 and December 2012 were collected from 38 affiliated hospitals of the Japanese Society of Myeloma. This survey included consecutively patients treated at the participating hospital. Treatment for each patient had been determined by the respective physician-in-charge. The data were compared with our historical data of 1208 patients,^11^ and the total of 3442 patients were analyzed. The diagnosis of MM was made according to the International Myeloma Working Group criteria and the clinical stage was determined based on the Durie & Salmon staging system (D&S) and/or the International Staging System (ISS).^12, 13^ Response to treatment was assessed according to the international uniform criteria.^14^ Fisher's exact test was used to compare differences between numerical values and the Mann–Whitney *U*-test was for categorical variables. Kaplan–Meier method was used to construct OS curves, and differences were analyzed by the log-rank test. The Cox proportional hazards model was applied for multivariate analysis to determine independent predictors associated with extended OS. This study was conducted in accordance with the institutional guidelines with approval of the Ethics Committee/Institutional Review Board of Tokushima Prefectural Central Hospital.

Baseline characteristics of the patients in each cohort are summarized in Table 1. Patient characteristics such as age, gender, type of M protein, D&S, hemoglobin, serum creatinine, calcium and the presence of chromosomal abnormality were not significantly different between the two cohorts. However, the percentages of patients with good performance status, advanced ISS and the elevated serum lactate dehydrogenase (LDH) were higher in the 2001–2012 cohort (*P*<0.001, *P*=0.02 and *P*<0.001, respectively). As for initial therapy, novel agents were not administered in the 1990–2000 cohort, and most of the patients (90.8%) were treated with conventional chemotherapy alone such as melphalan+prednisolone, and the remaining 9.2% patients were treated with ASCT after the induction therapy with vincristine, adriamycin and dexamethasone or other conventional regimens (Supplementary Table 1). In contrast, in the 2001–2012 cohort 48.1% of patients were treated with conventional chemotherapy, 19.6% with novel agent-containing regimens such as bortezomib+dexamethasone, 19.1% with conventional chemotherapy (mainly vincristine, adriamycin and dexamethasone)+ASCT, and the remaining 13.2% were treated with novel agent-based regimen (mainly bortezomib+dexamethasone)+ASCT. As for best response, although stringent complete response (sCR) was not assessable in the 1990–2000 cohort, the percentages of patients achieving a deep response such as complete response (CR) and very good partial response (VGPR) were higher in the 2001–2012 cohort (*P*<0.001, Supplementary Table 1).

The median OS was 60.6 months for the 2001–2012 cohort, which was significantly improved compared with that for the 1990–2000 cohort (38.9 months, *P*<0.0001, Figure 1a). According to the different age groups such as <50, 50–64, 65–74 and 75⩽ years, no significant difference was found among the age groups in the 1990–2000 cohort (Figure 1b). In contrast, in the 2001–2012 cohort the median OS was significantly extended in the younger age groups (97.3, 75.9, 55.4 and 45.5 months, respectively; and *P*-values between the adjacent groups were *P*=0.23, *P*<0.0001 and *P*=0.0007, respectively). When we divided the data of the 2001–2012 cohort into the 2001–2005 and 2006–2012, a significant improvement of the younger groups was observed again in the 2001–2005 cohort (the median OS were 74.4, 71.0, 52.7 and 52.6 months, respectively; and *P*-values were *P*=0.65, *P*=0.0012 and *P*=0.81, respectively; Supplementary Figure 1a), and the differences between the age groups became more prominent when confined to the 2006–2012 cohort (the median OS were not reached, 76.4, 57.3 and 42.6 months, respectively; and *P*-values were *P*=0.35, *P*=0.0021 and *P*<0.0001, respectively). This tendency of improving OS mostly in younger patients is consistent with the previous reports.^9, 10^ On the other hand, Kumar *et al.*^15^ have recently reported an improvement of OS in elderly patients (>65 years) rather than younger patients (⩽65 years) in the extended follow-up study at Mayo Clinic, suggesting that the widespread use of lenalidomide+dexamethasone as initial therapy was associated with improved OS in elderly patients.

As for OS according to the D&S, significant differences were found between stages I and III and stages II and III in both cohorts (both *P*<0.0001, Supplementary Figure 1b). As for OS according to the ISS, significant differences were observed between all the stages in both cohorts (stages I vs II (*P*<0.00001), I vs III (*P*<0.00001) and II vs III (*P*=0.0025) in the 1990–2000 cohort, and stages I vs II (*P*<0.0001) and II vs III (*P*<0.0001) in the 2001–2012 cohort; Supplementary Figure 1c]. According to chromosomal abnormality, the median OS of normal karyotype group was significantly longer than that of abnormal karyotype group in both cohorts (*P*<0.01 and *P*<0.0001, respectively; Supplementary Figure 1d). Thus, these results indicate that D&S, ISS and chromosomal abnormality are still important prognostic factors even in the era of novel agents and ASCT, and the prognosis of high-risk patients composed of advanced ISS and poor cytogenetics remains unimproved.

Regarding the outcome according to initial therapy, the median OS was 37.1 months for conventional chemotherapy group and 64.8 months for conventional chemotherapy+ASCT group in the 1990–2000 cohort (*P*<0.0001, Figure 1c). In the 2001–2012 cohort, the median OS of the four different treatment groups comprising conventional chemotherapy (1), novel agents (2), conventional chemotherapy+ASCT (3) and novel agents+ASCT (4) were 46.1, 62.5, 91.7 and 132.3 months, respectively (1 vs 2, *P*=0.0004; 1 vs 3, *P*<0.0001; 1 vs 4, *P*<0.0001; 2 vs 3, *P*<0.0001; 2 vs 4, *P*<0.0001; and 3 vs 4, *P*=0.26; Figure 1c). Thus, the survival benefit of ASCT seems more prominent in the recent cohort. According to the best response to first-line therapy, OS was significantly better in the deep response groups especially in the sCR of the recent cohort (CR vs VGPR, *P*=0.017; and VGPR vs PR, *P*<0.0001 in the 1990–2000 cohort; sCR vs CR, *P*=0.04; CR vs VGPR, *P*<0.01; and VGPR vs PR, *P*=0.0001 in the 2001–2012 cohort; Figure 1d). Furthermore, we evaluated the role of novel agents throughout the clinical course (Supplementary Figure 1e). The median OS was significantly improved in patients treated with novel agents either as first-line or subsequent treatment lines (second or third lines) compared with those treated without novel agents (66.8 and 39.4 months, respectively, *P*<0.00001).

In a multivariate analysis of the 2001–2012 cohort, variables studied were patient-baseline factors (age, gender and performance status), prognostic factors (hemoglobin, serum creatinine, calcium, LDH and karyotype), clinical stages (D&S and ISS) and treatment-related factors (initial therapy with novel agents and/or ASCT). In this analysis, poor performance status (*P*<0.0001), chromosomal abnormality (*P*<0.0001) and ISS stage III (*P*<0.0001) were significant poor prognostic factors for OS (Supplementary Table 2). Notably, initial therapies with novel agents and/or ASCT were significant favorable prognostic factors for OS (*P*=0.003 and *P*<0.0001, respectively).

In conclusion, we have demonstrated that the treatment modality has changed dramatically after the introduction of novel agents and ASCT in Japan, and the survival outcome has significantly improved especially in younger patients with low-risk and deep response to initial therapy. However, the survival benefit remains unmet in elderly and frail patients not suitable for these therapeutic modalities. Alternative approaches for better upfront management are still needed to further improve the outcome in elderly and high-risk patients with MM.

## Figures and Tables

**Figure 1:**
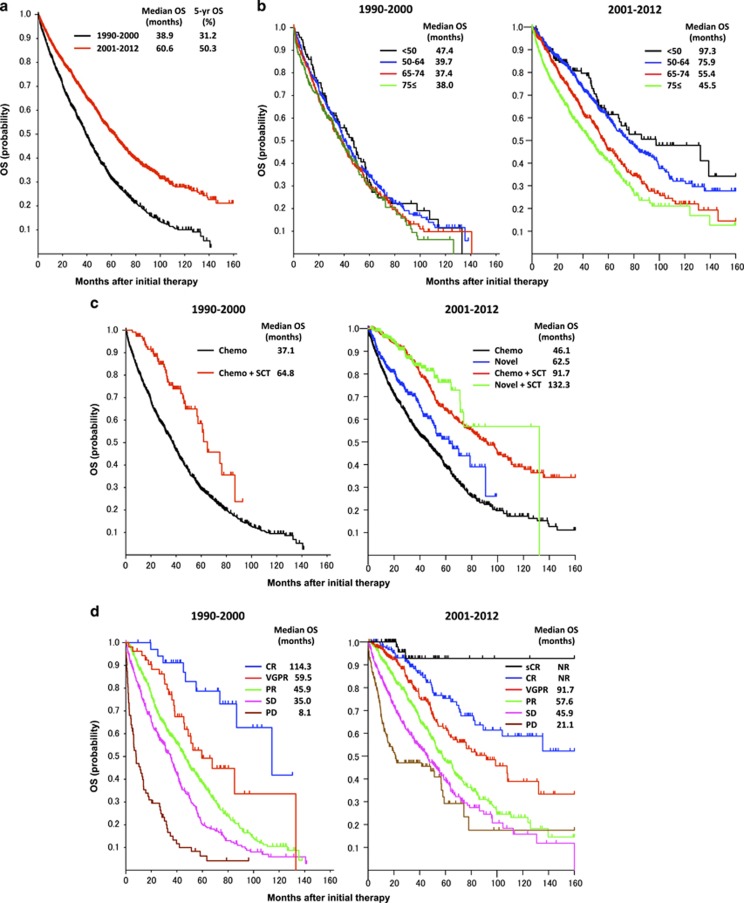
Overall survival according to the time periods (**a**), age groups (**b**), initial therapies (**c**), and best responses (**d**) in comparison of 1990–2000 vs 2001–2012.

**Table 1:** Patient characteristics

*Variable*	*1990–2000 Cohort (*n=*1208)*	*2001–2012 cohort (*n=*2234)*	P
Male/Female	628/580	1159/1075	0.96
Median age, years (range)	70 (31–96)	67 (26–96)	0.062
Age⩾75 (%)	354 (29.7)	723 (32.4)	
Performance status (%)			<0.001
0	221 (18.6)	305 (18.6)	
1	323 (27.2)	602 (36.7)	
2	286 (24.1)	396 (24.2)	
⩾3	358 (30.1)	336 (20.5)	
Unknown	20	595	
M protein type (%)			0.63
IgG	693 (58.0)	1329 (60.0)	
IgA	259 (21.7)	415 (18.7)	
IgD	42 (3.5)	63 (2.8)	
Light chain	155 (13.0)	347 (15.6)	
Others	44 (3.7)	20 (0.9)	
Non-secretory	1 (0.1)	44 (2.0)	
Unknown	14	16	
Durie & Salmon stage (%)			0.16
I	111 (9.5)	180 (8.4)	
II	316 (27.1)	546 (25.4)	
III	741 (63.4)	1424 (66.2)	
Unknown	40	84	
ISS stage (%)			0.02
I	290 (33.3)	509 (26.5)	
II	293 (33.6)	750 (39.1)	
III	288 (33.1)	660 (34.4)	
Unknown	337	315	
Hb (%)			0.06
Normal	509 (43.3)	941 (47.3)	
Low (<10 g/dl)	667 (56.7)	1048 (52.7)	
Unknown	32	245	
Serum Cr (%)			0.92
Normal	990 (84.7)	1671 (84.5)	
High (>2 mg/dl)	179 (15.3)	307 (15.5)	
Unknown	39	256	
Serum Ca (%)			0.35
Normal	1032 (90.9)	1631 (88.9)	
High (>11 mg/dl)	103 (9.1)	204 (11.1)	
Unknown	73	399	
Serum LDH (%)			<0.001
Normal	971 (82.3)	1434 (73.1)	
High (>normal upper limit)	209 (17.7)	528 (26.9)	
Unknown	28	272	
Karyotype (%)			0.15
Normal	228 (82.9)	1149 (77.4)	
Abnormal	47 (17.1)	335 (22.6)	
Unknown	933	750	

Abbreviations: ISS, international staging system; LDH, lactate dehydrogenase.
