# Supplementary material for: Trends of survival in patients with multiple myeloma in Japan: a multicenter retrospective collaborative study of the Japanese Society of Myeloma
Source: Blood Cancer J. 2015 Sep 18;5(9):e349–. doi: 10.1038/bcj.2015.79 (PMC4648525; doi:10.1038/bcj.2015.79)
Supplement: Supplementary Figure 1 Legend [file bcj201579x1.doc]

**Supplementary Figure legends**

**Supplementary Figure 1.**

Overall survival according to the age groups in comparison of 2001-2005 vs 2006-2012 (a). Overall survival according to Durie and Salmon stage (b), ISS stage (c), chromosomal abnormality (d), and the use of novel agents at any time during the clinical course (e) in comparison of 1990-2000 vs 2001-2012.
