# Supplementary material for: Trends of survival in patients with multiple myeloma in Japan: a multicenter retrospective collaborative study of the Japanese Society of Myeloma
Source: Blood Cancer J. 2015 Sep 18;5(9):e349–. doi: 10.1038/bcj.2015.79 (PMC4648525; doi:10.1038/bcj.2015.79)

Supplementary Figure 1

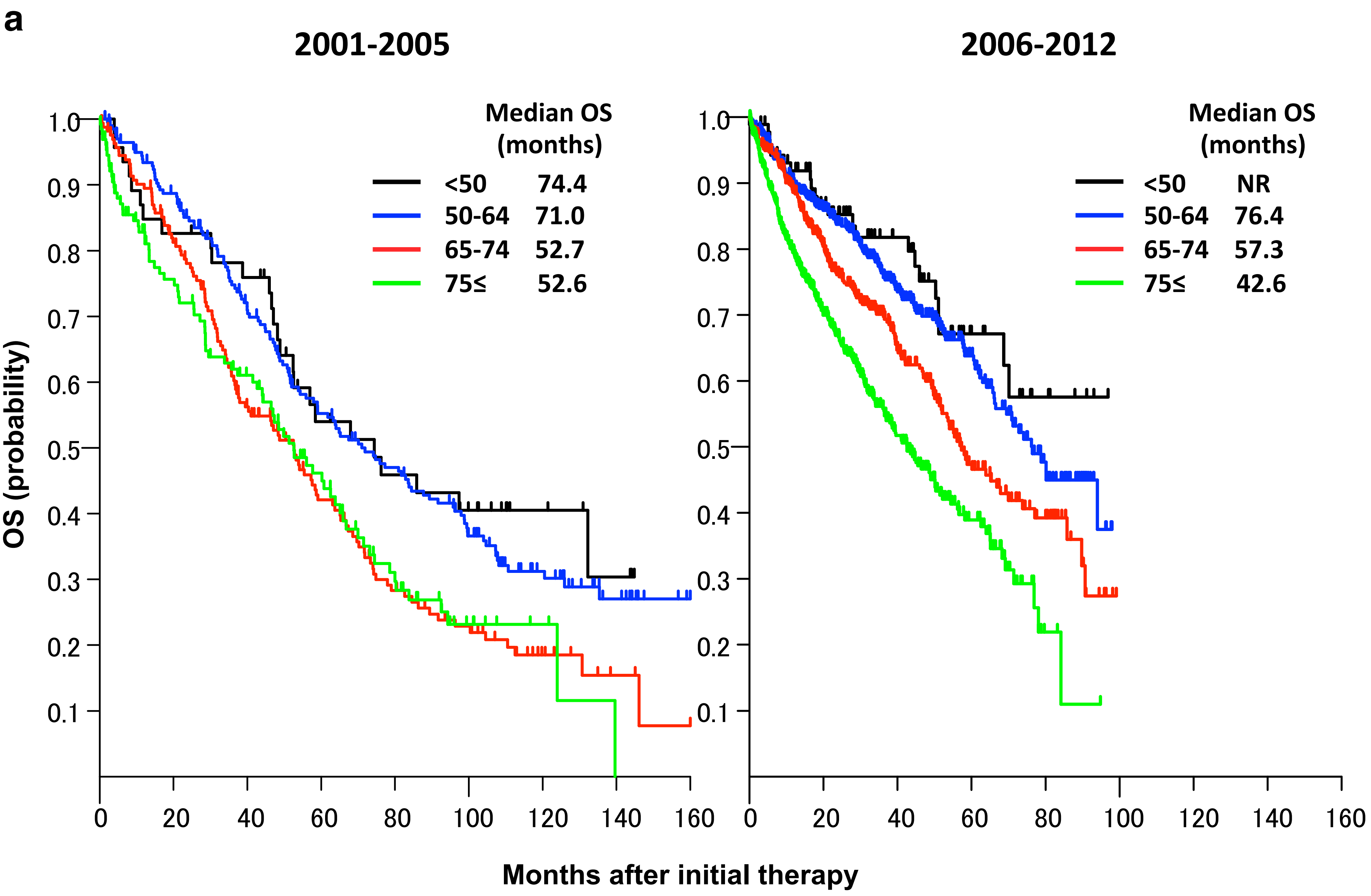

Supplementary Figure 1

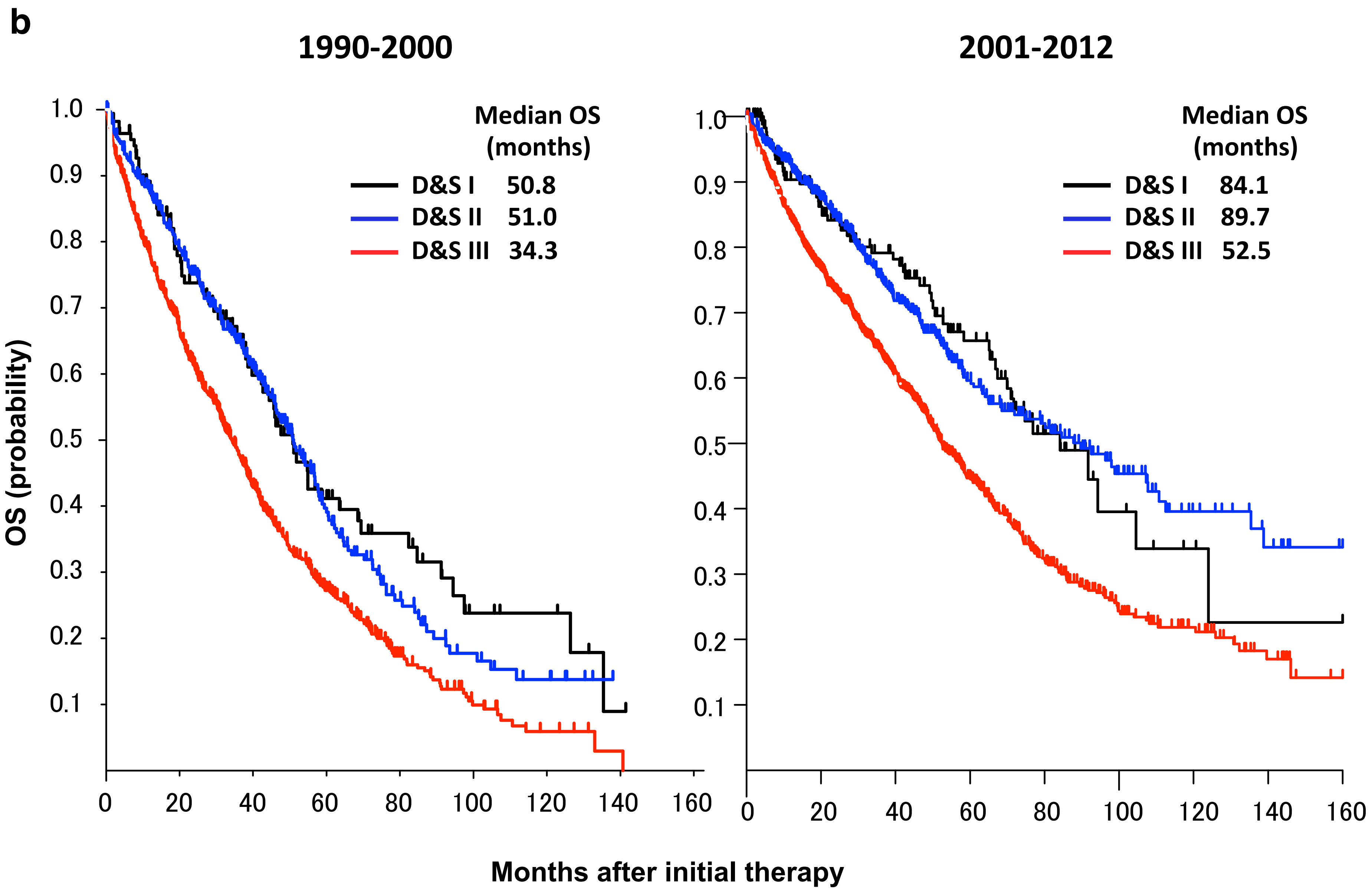

Supplementary Figure 1

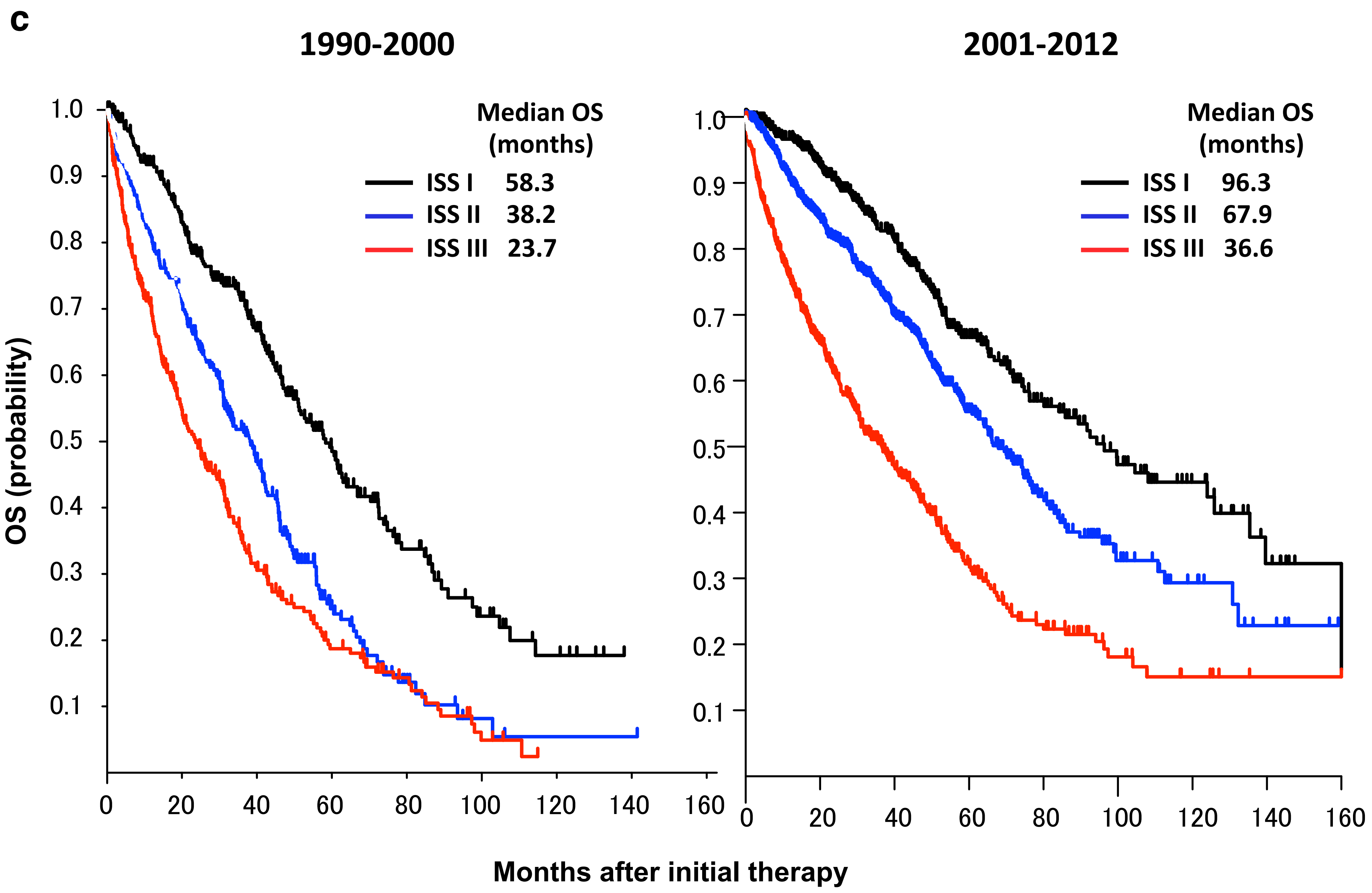

Supplementary Figure 1

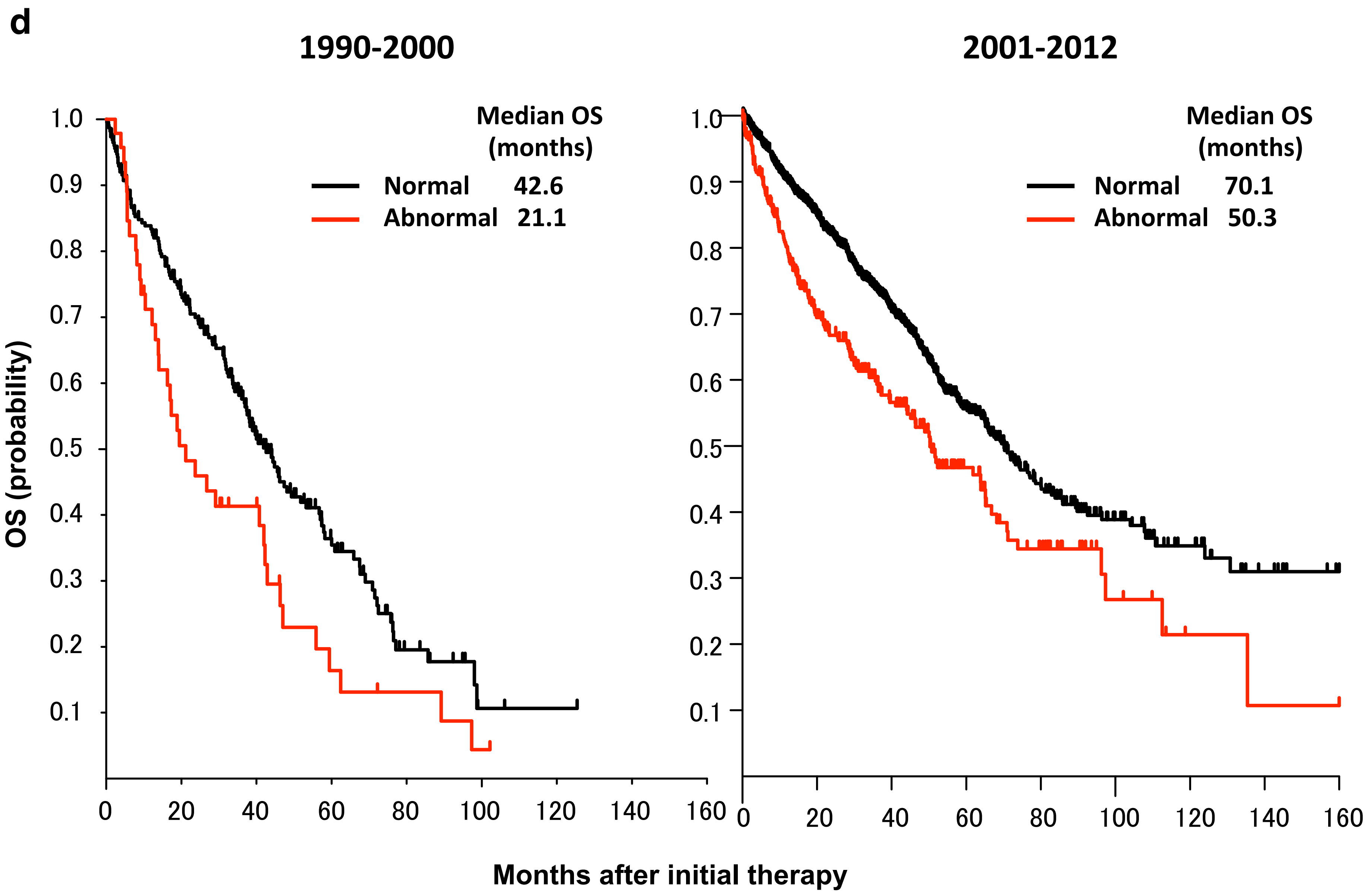

Supplementary Figure 1

e

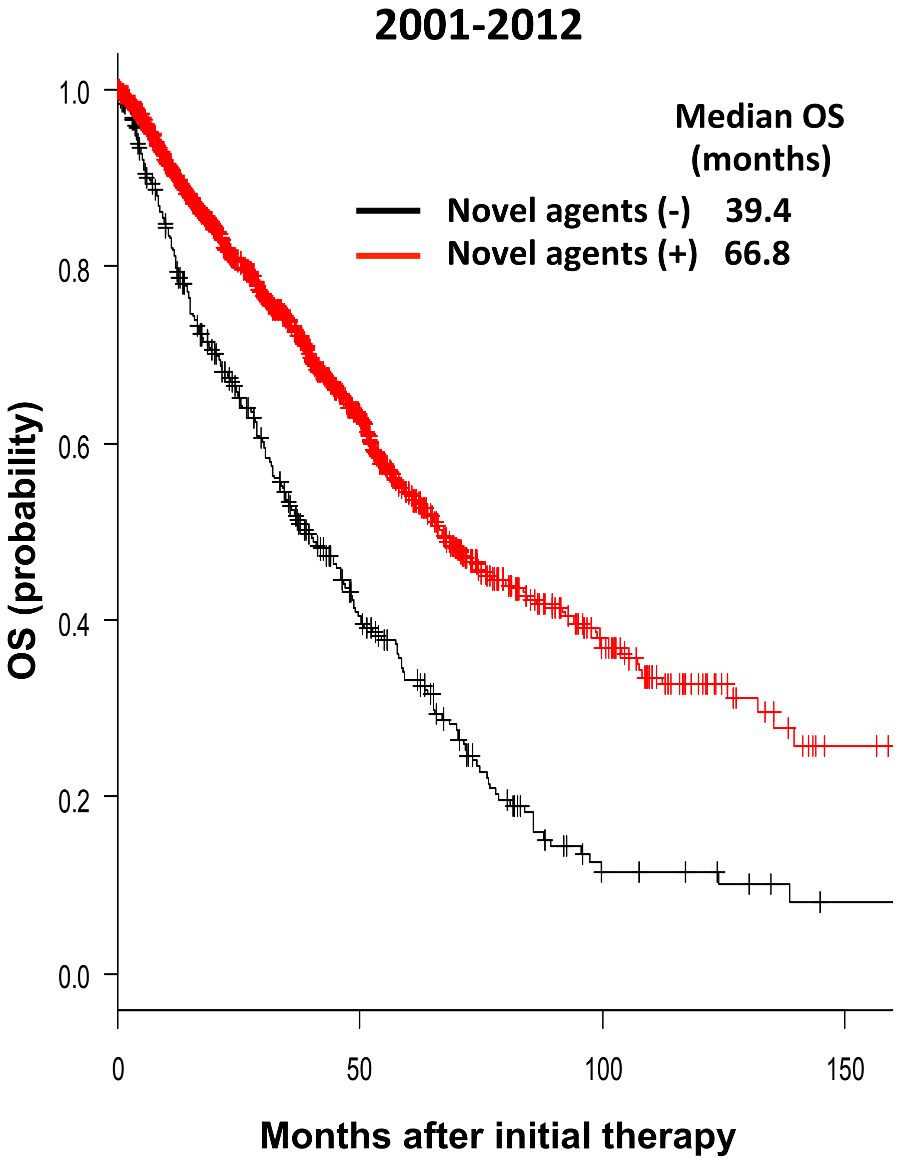

Supplement: Supplementary Figure 1 [file bcj201579x2.pdf]
