# Supplementary material for: Trends of survival in patients with multiple myeloma in Japan: a multicenter retrospective collaborative study of the Japanese Society of Myeloma
Source: Blood Cancer J. 2015 Sep 18;5(9):e349–. doi: 10.1038/bcj.2015.79 (PMC4648525; doi:10.1038/bcj.2015.79)
Supplement: Supplementary Table 1 [file bcj201579x3.doc]

**Supplementary Table 1. Initial therapy and best response**

| Variable | 1990-2000 cohort  (n=1,208) | 2001-2012 cohort  (n=2,234) |
| --- | --- | --- |
| Conventional Chemotherapy (%)  MP  MMCP  VAD  VMCP  ROAD  DEX  Others | 321 (29.3)  150 (13.7)  98 (8.9)  64 (5.8)  31 (2.8)  0 (0.0)  433 (39.5) | 558 (52.4)  4 (0.4)  182 (17.1)  0 (0.0)  9 (0.8)  167 (15.7)  145 (13.6) |
| Novel agents (%)  BD  VMP  VCD  TD  MPT  Others | 0  0  0  0  0  0 | 260 (59.8)  83 (19.1)  27 (6.2)  24 (5.5)  20 (4.6)  21 (4.8) |
| Chemotherapy + ASCT (%)  VAD  DEX  Others | 66 (59.5)  0 (0.0)  45 (40.5) | 317 (74.8)  45 (10.6)  62 (14.6) |
| Novel agents + ASCT (%)  BD  VCD  PAD  TD  Others | 0  0  0  0  0 | 216 (74.0)  47 (16.1)  12 (4.1)  10 (3.4)  7 (2.4) |
| Best response (%)  sCR  CR  VGPR  PR  SD  PD | NA  36 (3.2)  53 (4.7)  573 (51.0)  390 (34.7)  72 (6.4) | 74 (4.6)  153 (9.5)  271 (16.8)  594 (36.8)  467 (29.0)  53 (3.3) |

MP: melphalan + prednosolone; MMCP: ranimustine + melphalan + cyclophosphamide + prednisolone; VAD: vincristine + adriamycin + dexamethasone; VMCP: vincristine + melphalan + cyclophosphamide + prednisolone; ROAD: ranimustine + vincristine + melphalan + dexamethasone; DEX: dexamethasone; BD: bortezomib + dexamethasone; VMP: bortezomib + melphalan + prednisolone; VCD: bortezomib + cyclophosphamide + dexamethasone; TD: thalidomide + dexamethasone; MPT: melphalan + prednisolone + thalidomide; PAD: adriamycin + bortezomib + dexamethasone; ASCT: autologous stem cell transplantation; NA: not applicable
