# Supplementary material for: Trends of survival in patients with multiple myeloma in Japan: a multicenter retrospective collaborative study of the Japanese Society of Myeloma
Source: Blood Cancer J. 2015 Sep 18;5(9):e349–. doi: 10.1038/bcj.2015.79 (PMC4648525; doi:10.1038/bcj.2015.79)
Supplement: Supplementary Table 2 [file bcj201579x4.doc]

**Supplementary Table 2. Multivariate analysis for overall survival**

| Variable | RR | 95% CI | P |
| --- | --- | --- | --- |
| Age (≥65 years) | 1.258 | 0.986 – 1.605 | 0.065 |
| Gender (Male) | 0.779 | 0.652 – 0.932 | 0.006 |
| Performance status (≥2) | 1.696 | 1.363 – 2.111 | <0.0001 |
| Hb (<10 g/dl) | 0.906 | 0.719 – 1.140 | 0.398 |
| Serum Cr (>2 mg/dl) | 0.961 | 0.716 – 1.289 | 0.792 |
| Serum Ca (>12 mg/dl) | 1.423 | 1.012 – 2.005 | 0.042 |
| Serum LDH (above the ULN) | 1.326 | 1.075 – 1.635 | 0.008 |
| Karyotype (abnormal) | 1.619 | 1.272 – 2.061 | <0.0001 |
| Durie and Salmon (Stage III) | 1.543 | 1.183 – 2.013 | 0.001 |
| ISS (Stage III) | 2.262 | 1.788 – 2.861 | <0.0001 |
| Initial therapy with novel agents* | 0.636 | 0.474 – 0.853 | 0.003 |
| Initial therapy with ASCT* | 0.459 | 0.350 – 0.603 | <0.0001 |

RR, relative risk; CI, confidence interval

ULN, upper limit of the normal range

*Initial therapy was categorized as chemotherapy (1), novel agents (2), chemotherapy + ASCT (3), and novel agents + ASCT (4).
