# Supplementary material for: Trends of survival in patients with multiple myeloma in Japan: a multicenter retrospective collaborative study of the Japanese Society of Myeloma
Source: Blood Cancer J. 2015 Sep 18;5(9):e349–. doi: 10.1038/bcj.2015.79 (PMC4648525; doi:10.1038/bcj.2015.79)
Supplement: Supplementary Information [file bcj201579x5.doc]

**Supplementary Appendix**

The following Japanese Society of Myeloma (JSM) institutions and investigators participated in this study: Tadao Ishida, Department of Gastroenterology, Rheumatology, and Clinical Immunology, Sapporo Medical University School of Medicine, Sapporo; Takahiro Kobayashi, Takaya Yamashita, Naoto Takahashi, Department of Hematology, Nephrology and Rheumatology, Akita University Hospital, Akita; Hiroshi Handa, Takayuki Saitoh, Hirokazu Murakami, Department of Hematology, Gunma University Hospital, Maebashi; Atsushi Isoda, Morio Matsumoto, Morio Sawamura, Department of Hematology, National Hospital Organization Nishigunma National Hospital, Shibukawa; Eishi Nogiwa, Department of Internal Medicine, Usui Hospital, Yasunaka: Chiaki Nakaseko, Department of Hematology, Chiba University Hospital, Chiba; Fumihiko Nakamura, Department of Hematology and Oncology, Graduate School of Medicine, The University of Tokyo, Tokyo; Go Yamamoto, Department of Hematology, Toranomon Hospital, Tokyo; Kenshi Suzuki, Department of Hematology, Japanese Red Cross Medical Center, Tokyo; Shotaro Hagiwara, Division of Hematology, Department of Internal Medicine, National Medical Center for Global Health and Medicine, Tokyo; Naoki Takezako, Department of Hematology, National Hospital Organization National Disaster Medical Center, Tokyo; Masao Hagihara, Department of Hematology, Eiju General Hospital, Tokyo; Shinya Okuda, Department of Hematology and Oncology, JR Tokyo General Hospital, Tokyo; Naoto Tomita, Department of Internal Medicine and Clinical Immunology, Yokohama City University Hospital, Yokohama; Tomonori Nakazato, Department of Hematology, Yokohama Municipal Citizen's Hospital, Yokohama; Akiko Negoro, Department of Hematology, Japan Labor Health and Welfare Organization Yokohama Rosai Hospital, Yokohama; Takaaki Cho, Department of Medicine, Niigata Cancer Center Hospital, Niigata; Junji Ito, Department of Clinical Laboratory, Nagoya City Midori General Hospital; Nagoya; Hiroshi Kosugi, Department of Hematology, Ogaki Municipal Hospital, Ogaki; Hiroyuki Takamatsu, Department of Hematology, Kanazawa University Graduate School of Medical Science, Kanazawa; Hiroyuki Takamatsu, Department of Hematology, NTT WEST Kanazawa Hospital, Kanazawa; Tatsuharu Ohno, Division of Hematology and Immunology, Department of Internal Medicine, Ohtsu Red Cross Hospital, Ohtsu; Junya Kuroda, Department of Hematology and Oncology, Kyoto Prefectural University of Medicine, Kyoto; Chihiro Shimazaki, Department of Hematology, Japan Community Healthcare Organization Kyoto-Kuramaguchi Medical Center, Kyoto; Nobumasa Inoue, Department of Internal Medicine, National Hospital Organization Osaka Medical Center, Osaka; Toru Murayama, Department of Hematology, Hyogo Cancer Center, Akashi; Jun Konishi, Kazutaka Sunami, Department of Hematology, National Hospital Organization Okayama Medical Center, Okayama; Yoshiaki Kuroda, Department of Hematology, Hiroshima University Hospital, Hiroshima; Mitsuhiro Itagaki, Hideki Asaoku, Department of Hematology, Hiroshima Red Cross Hospital, Hiroshima; Takaaki Miyake, Department of Hematology, Shimane University Hospital, Izumo; Toshio Wakayama, Department of Hematology, Shimane Prefectural Central Hospital, Izumo; Takeshi Harada, Masahiro Abe, Department of Hematology, Tokushima University Hospital, Tokushima; Shuji Ozaki, Etsuko Sekimoto, Hironobu Shibata, Toshio Shigekiyo, Department of Hematology, Tokushima Prefectural Central Hospital, Tokushima; Toshihiro Hashimoto, Department of Internal Medicine, Tokushima Municipal Hospital, Tokushima; Yasushi Takamatsu, Division of Medical Oncology, Hematology, and Infectious Disease, Department of Internal Medicine, Fukuoka University, Fukuoka; Naokuni Uike, Department of Hematology, National Kyushu Cancer Center, Fukuoka; Hiroyuki Hata, Department of Immunology and Hematology, Faculty of Life Sciences, Graduate School of Health Sciences, Kumamoto University, Kumamoto; Naoko Harada, Department of Hematology, National Hospital Organization Kumamoto Medical Center, Kumamoto, Japan.
